# Significant incidental findings in 9-year old children undergoing cardiovascular magnetic resonance imaging for research

**DOI:** 10.1186/1532-429X-16-S1-T8

**Published:** 2014-01-16

**Authors:** Jennifer A Bryant, Keith Godfrey, Mark Hanson, Charles Peebles

**Affiliations:** 1 Cardiothoracic Radiology, University Hospital Southampton NHS Foundation Trust, Southampton, UK; 2 MRC Lifecourse Epidemiology Unit, Southampton, UK; 3 NIHR Southampton Biomedical Research Centre, University of Southampton and University Hospital Southampton NHS Foundation Trust, Southampton, UK; 4 Institute of Developmental Sciences, University of Southampton, Southampton, UK

## Background

The management of incidental findings in research imaging is a contentious issue. The imaging field of view in cardiovascular magnetic resonance imaging (CMR) studies often extends well beyond the cardiac anatomy, particularly on initial scout views. In clinical CMR there is limited data on incidental extra-cardiac findings in adults and even less in children. In research studies we are unaware of any data on incidental findings (cardiac or extra-cardiac) involving CMR of children. The aim of this study was to report on significant incidental findings noted in a research cohort of healthy children undergoing CMR.

## Methods

A comprehensive study of cardiovascular structure and function was performed on 350 healthy children aged 9 years scanned as part of a study of developmental influences on cardiovascular structure and function. A consultant Radiologist reviewed all images. Only potentially significant cardiac or extra-cardiac findings were recorded. Findings were considered significant if they required further investigation or management.

## Results

**Figure 1:**
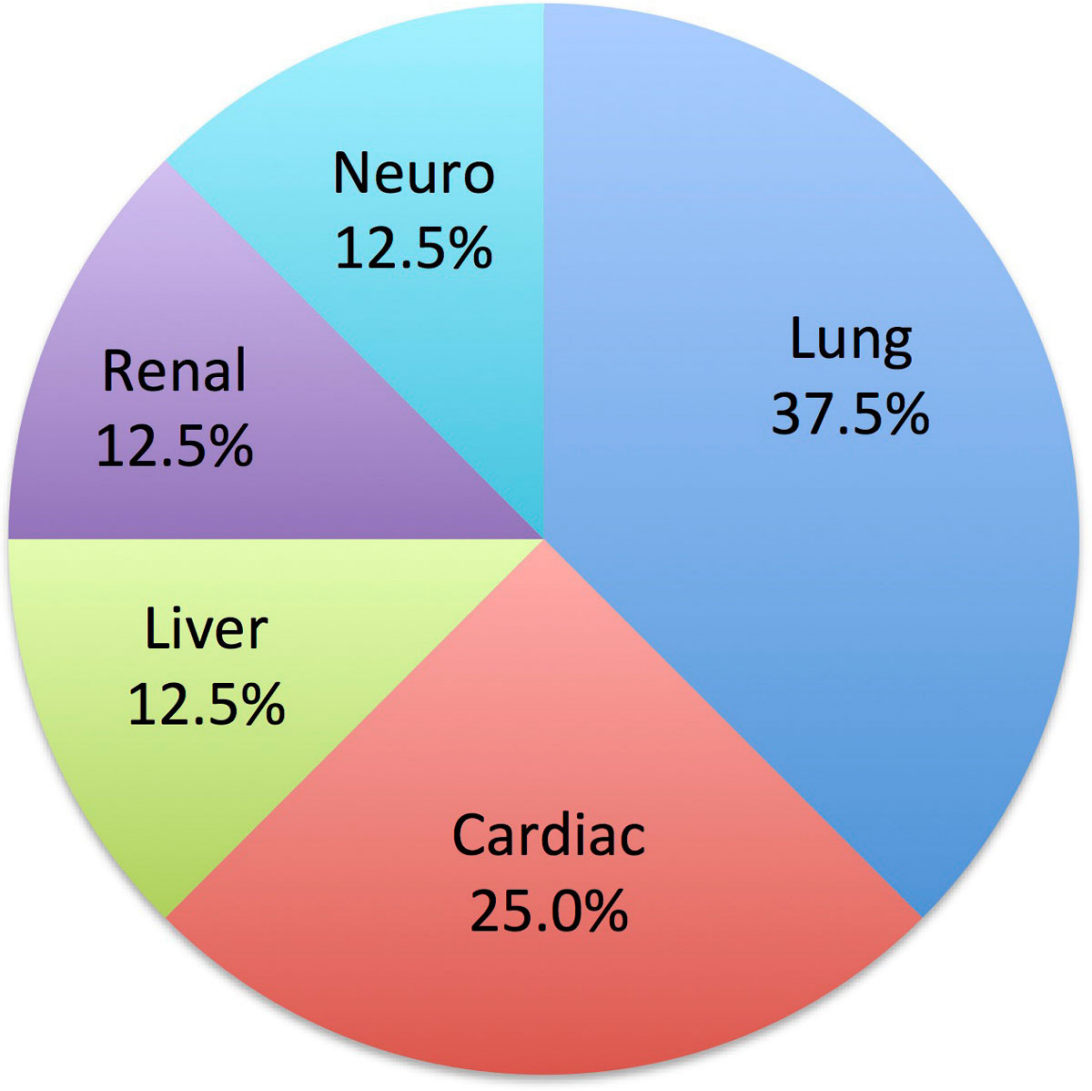
**Percentage significant incidental findings by body part**.

Significant incidental findings were identified in 8 children: 2 cardiac (atrio-ventricular septal defect (AVSD), bicuspid aortic valve) and 6 extra-cardiac (Figure 1). All of these findings required further imaging or follow-up. Three chest findings required chest x-ray and clinical follow-up; the bicuspid valve was referred for echo assessment and follow-up; the AVSD required urgent Cardiology assessment and ultimately surgical correction; clinical follow-up was recommended for a pelvic kidney; liver and brain lesions required further cross-sectional imaging.

## Conclusions

In 2.3% of our cohort there were significant incidental findings (1.7% extra-cardiac). Despite the age group this figure is similar to the highly significant extra-cardiac findings reported in similar studies of adults. Incidental findings are an important consideration in imaging for research. It is advisable that a Radiologist reviews all imaging, and that clear follow-up pathways are established to manage any potential clinically significant findings.

## Funding

This work was supported by grants from the Medical Research Council, British Heart Foundation, European Union's Seventh Framework Programme (FP7/2007-2013, project Early Nutrition under grant agreement n°289346) and NIHR Southampton Biomedical Research Centre, University of Southampton and University Hospital Southampton NHS Foundation Trust.

